# The association between *LRP1* and chylomicron uptake after the ingestion of a high-fat meal

**DOI:** 10.1186/1753-6561-6-S6-P8

**Published:** 2012-10-01

**Authors:** AC Frazier-Wood, EK Kabagambe, MK Wojczynski, IB Borecki, HK Tiwari, CE Smith, JM Ordovas, DK Arnett

**Affiliations:** 1 Division of Epidemiology, Genetics and Environmental Sciences, University of Texas School of Public Health, Houston, TX, USA; 2 Department of Epidemiology, University of Alabama at Birmingham, Birmingham, AL, USA; 3 Nutrition Obesity Research Center, University of Alabama at Birmingham, School of Public Health, Birmingham, AL 35294, USA; 4 Division of Statistical Genomics, Department of Genetics, Washington University, School of Medicine, 4444 Forest Park Boulevard - Box 8506, St Louis, MO 63108, USA; 5 Section on Statistical Genetics, University of Alabama at Birmingham, School of Public Health, Birmingham, AL 35294, USA; 6 Nutrition and Genomics Laboratory, Jean Mayer-US Department of Agriculture Human Nutrition Research Center on Aging, Tufts University, Boston, MA, USA; 7 The Department of Epidemiology and Population Genetics. Centro Nacional Investigación Cardiovasculares (CNIC) Madrid, Spain; 8 IMDEA Food, Madrid, Spain

## Background

Evidence suggests that patients find the use of genetic information alongside dietary strategies in the treatment of obesity more understandable and more useful than general dietary advice alone [1]. But, as yet, specific variants that associate with individual responses to specific dietary factors in humans have yet to be conclusively identified. Chylomicrons are secreted by the small intestine after the intake of dietary fat, when they are responsible for the transport of exogenous lipids into the tissue. *In vitro* studies suggest that the low density lipoprotein receptor related protein 1 (*LRP1*) gene plays a role in the secondary uptake of chylomicrons, with *LRP1* knockout mice showing decreased chylomicron clearance. Whether this is true in humans is unknown. We aimed to determine whether genetic variants in *LRP1* are associated with postprandial chylomicron uptake in humans.

## Materials and methods

We used a general population cohort of 744 men and women (mean age ± SD, 49 ± 16 years) participating in the Genetics of Lipid Lowering Network Study. Chylomicrons were measured by nuclear resonance spectroscopy before, and 3.5 and 6 h after, participants were given an oral fat challenge (700 kcal/m^2^ body surface area, at 83% calories from fat). SNPs in the *LRP1* gene (*n* = 26) were initially tested for association with changes in chylomicron concentrations between 3.5 and 6 h after the high-fat meal using mixed linear models adjusted for age, sex, study site and pedigree, assuming an additive genetic model. Linkage disequilibrium (LD) was calculated using Haploview software [2]. Subsequently, a gene-based test statistic which adjusted for the LD across the SNPs was calculated using VEGAS [3].

## Results

Of 26 *LRP1* SNPs, 11 were significantly associated with the change in chylomicron concentration after a false discovery rate correction for multiple testing (*Q*<0.05), across two haplotype blocks. The subsequent gene-based test, corrected for LD and multiple testing, was also significant (*P* = 0.01).

## Conclusions

These results implicate the role of *LRP1* in postprandial lipoprotein uptake and/or clearance. Given the role of chylomicron clearance in subsequent fat accumulation, if these results are replicated, this information may eventually help tailor dietary advice, aimed at reducing BMI, in the pursuit of personalized medicine paradigm in the treatment of obesity.
